# Re-Definition of the Epidemiology of Cardiac Amyloidosis

**DOI:** 10.3390/biomedicines10071566

**Published:** 2022-06-30

**Authors:** Maddalena Rossi, Guerino Giuseppe Varrà, Aldostefano Porcari, Riccardo Saro, Linda Pagura, Andrea Lalario, Franca Dore, Rossana Bussani, Gianfranco Sinagra, Marco Merlo

**Affiliations:** 1 Center for Diagnosis and Treatment of Cardiomyopathies, Cardiovascular Department, Azienda Sanitaria Universitaria Giuliano-Isontina (ASUGI) and University of Trieste, 34149 Trieste, Italy; maddalenarossi93@gmail.com (M.R.); giuseppevarra@hotmail.it (G.G.V.); aldostefanoporcari@gmail.com (A.P.); riccardo.saro@gmail.com (R.S.); lindapagura91@gmail.com (L.P.); lalarioandrea@gmail.com (A.L.); gianfranco.sinagra@asugi.sanita.fvg.it (G.S.); 2 Department of Nuclear Medicine, Azienda Sanitaria Universitaria Giuliano-Isontina (ASUGI) and University of Trieste, 34149 Trieste, Italy; franca.dore@asugi.sanita.fvg.it; 3 Center for Diagnosis and Treatment of Cardiomyopathies, Cardiothoracic Department, Institute of Pathological Anatomy and Histology, Azienda Sanitaria Universitaria Giuliano-Isontina (ASUGI), University of Trieste, 34149 Trieste, Italy; bussani@units.it

**Keywords:** cardiac amyloidosis, epidemiology, red flags, heart failure, hypertrophy, carpal tunnel syndrome, bone scintigraphy

## Abstract

The epidemiology of cardiac amyloidosis (CA), traditionally considered a rare and incurable disease, has changed drastically over the last ten years, particularly due to the advances in diagnostic methods and therapeutic options in the field of transthyretin CA (ATTR-CA). On the one hand, the possibility of employing cardiac scintigraphy with bone tracers to diagnose ATTR-CA without a biopsy has unveiled the real prevalence of the disease; on the other, the emergence of effective treatments, such as tafamidis, has rendered an early and accurate diagnosis critical. Interestingly, the following subgroups of patients have been found to have a higher prevalence of CA: elderly subjects > 75 years, patients with cardiac hypertrophy hospitalized for heart failure with preserved ejection fraction, subjects operated on for bilateral carpal tunnel syndrome, patients with cardiac hypertrophy not explained by concomitant factors and individuals with aortic valve stenosis. Many studies investigating the prevalence of CA in these particular populations have contributed to rewriting the epidemiology of the disease, increasing the awareness of the medical community for a previously underappreciated condition. In this review, we summarized the latest evidence on the epidemiology of CA according to the different clinical settings typically associated with the disease.

## 1. Introduction

Amyloidosis is a heterogeneous group of acquired or hereditary, localized or systemic diseases, caused by the extracellular deposition of insoluble fibrils derived from misfolded proteins [1]. Cardiac amyloidosis (CA) is the paradigm of restrictive cardiomyopathy and an emerging cause of morbidity and mortality worldwide [2]. Although there are more than 30 known amyloidogenic precursors, the forms of CA most frequently encountered in clinical practice are caused by the deposition of monoclonal immunoglobulin light chains (AL) or transthyretin (ATTR), either in its hereditary (ATTRv) or acquired (ATTRwt) form [3]. The epidemiology of the disease, classically considered rare and incurable, has radically changed in the last few years due to significant advances in diagnostic and therapeutic strategies, arousing growing interest in the scientific community [4].

The most significant innovations concern ATTR-CA, whose diagnostic work-up has been deeply transformed by the possibility of reaching a non-biopsy diagnosis through cardiac scintigraphy with bone tracers. This technique represents the cornerstone of the algorithm proposed by Gillmore et al. in 2016 and validated in a large cohort of patients with CA, where it has demonstrated sensitivity and specificity values of 99% and 86%, respectively, with a positive predictive value close to 100% if associated with serum/urinary immunofixation and the determination of free light chains ruling out the presence of monoclonal proteins [5]. Consequently, the diagnoses of ATTR-CA have increased exponentially and patients have been diagnosed earlier in the disease course, also because of an increased awareness of CA among physicians of many specialties.

Indeed, it is now well-known that amyloidosis is a systemic disease that needs multidisciplinary approaches for diagnosis and treatment. Until recently, ATTRv amyloidosis had been considered a disease of neurological interest because neuropathic symptoms predominated, whereas advances in diagnostic techniques revealed the presence of patients with cardiomyopathy as a predominant feature [6]. On the other hand, ATTRwt amyloidosis was considered a disease in the field of cardiology but recent studies have suggested that many patients with ATTRwt amyloidosis present tenosynovial tissue complications, such as carpal tunnel syndrome (CTS), spinal canal stenosis, brachial biceps tendon rupture and sensorimotor polyneuropathy with autonomic symptoms and orthostatic intolerance [6].

Therefore, the necessity for the close cooperation of multiple departments has increased to facilitate earlier diagnoses and better management of this group of patients, necessitating an awareness among cardiologists, neurologists and orthopedists.

A prospective multicentric study (AC-TIVE study) conducted in Italy investigated the prevalence of CA among patients with echocardiographic red flags and evaluated their diagnostic accuracy. The study design included the following two phases: Phase 1, of >5000 unselected patients, with an age of ≥55 years undergoing echocardiographic evaluation, demonstrated the presence of at least one echocardiographic red flag of CA in 7% of cases. Phase 2 involving a multiparametric diagnostic algorithm including bone scintigraphy and the search for a monoclonal protein to estimate disease prevalence among patients with ≥1 echocardiographic red flag, demonstrated a definite diagnosis of CA in 62 patients with a prevalence of 29% (51 ATTR-CA, 11 AL-CA). Apical sparing or a combination of ≥2 other echocardiographic red flags, excluding interatrial septum thickness, provided a diagnostic accuracy of >70%. ATTR-CA diagnosis increased with age from 12% at <70 years to 33% in patients ≥ 80 years. Compared to non-CA patients, CA patients were more frequently referred for echocardiography for heart failure (HF) and unexplained left ventricular hypertrophy (LVH) [7,8]. Based on these results, it appears important to change our approach to patients undergoing an echocardiogram, in order to systematically search for CA red flags, regardless of the exam indication; in this way, the echocardiography laboratory will become essential for obtaining an early diagnosis.

These advances, in association with the current availability of “disease-modifying” therapies, such as tafamidis [9], contribute to the increasing recognition of CA in a wide range of clinical scenarios. Whilst, according to available estimates, CA is considered a rare disease occurring in fewer than 5 people in 10,000, epidemiological data mostly derive from single-centre studies or population registries [10,11]. In the last few years, the following subgroups of patients have been found to have a higher prevalence of CA: elderly subjects > 75 years, patients with LVH hospitalized for heart failure with preserved ejection fraction (HFpEF), subjects operated on for bilateral carpal tunnel syndrome (CTS), patients with cardiac hypertrophy not explained by concomitant factors and individuals with aortic valve stenosis (AS). However, the real prevalence of the disease remains unknown and studies are needed in order to address the issue.

Moreover, many grey areas that physicians face during their daily clinical practice remain to be explored, both in terms of diagnostic tools, accurate prognostic stratification and best therapeutic options, i.e., the optimal “supportive” treatments for CA patients with HF [12].

**Figure 1:**
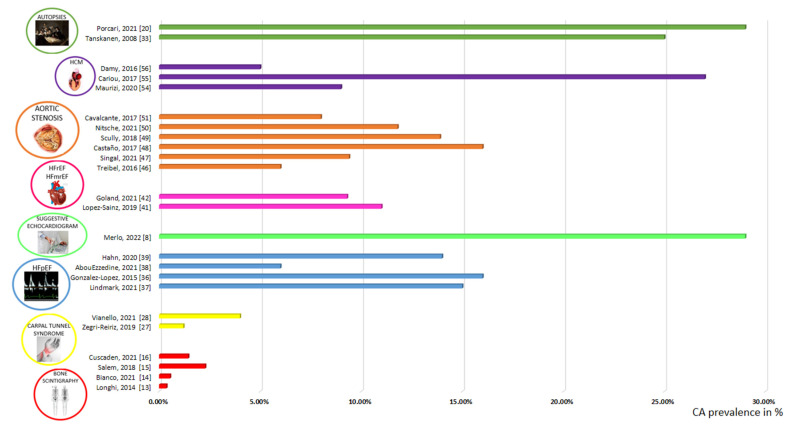
Prevalence of CA in different screening studies [8,13,14,15,16,20,27,28,33,36,37,38,39,41,42,46,47,48,49,50,51,54,55,56].

In this review, we summarized the latest evidence on the epidemiology of CA in different clinical settings (Figure 1).

## 2. Unveiling the Real Prevalence of CA: Reports from Scintigraphy with Bone Tracers Performed for Non Cardiac Reasons and Autopsies

What really has changed the epidemiology of amyloidosis in the last few years is the possibility to achieve a non-invasive diagnosis, according to the Gillmore algorithm [5], whose core element is represented by scintigraphy with bone tracers. Many studies reporting the prevalence of myocardial uptake such as an incidental finding during bone scintigraphy, have recently contributed to uncovering that CA is more common than previously thought. In 2014, Longhi et al. retrospectively analyzed 12,400 99mTc-3,3-diphosphono-1,2-propanodicarboxylic acid (99mTc-DPD) scintigraphy procedures for oncologic or rheumatologic reasons, finding unexpected myocardial tracer uptake in 0,36% of subjects, with the prevalence increasing progressively with age, peaking at 1.4% in men older than 80 years [13]. All of those who underwent subsequent cardiological evaluation showed an unexplained/out of proportion increase in the left ventricle (LV) wall thickness, and were then diagnosed with ATTR-CA (5 confirmed by EMB) [13]. These data were confirmed in a following study, which reported a 0.54% prevalence of myocardial uptake at bone scintigraphy in an all-comers population. All these patients showed a degree of heart involvement in echocardiography, with at least a mildly abnormal LV wall thickness and a moderately abnormal indexed LV mass [14]. Analyzing data from a cohort of individuals > 75 years with no previous clinical suspicion of ATTR, Salem et al. reported a 2.28% prevalence of cardiac uptake, reaching 13.9% and 2.7% in males and females ≥ 85 years [11]. Interestingly, they collected data about HF hospitalizations rates, describing that, after adjusting for age and gender, cardiac uptake was associated with a significantly higher risk of HF hospitalization [15]. Later, Cuscaden et al. reported that in a cohort of 6918 patients undergoing bone scans for non-cardiac indications, using 99mTc-hydroxymethylene diphosphonate (HMDP), 1.44% males and 0.17% females ≥ 65 showed a pattern consistent with ATTR-CA, also confirmed by a single-photon emission computed tomography (SPECT) analysis. As expected, prevalence increased with age, reaching 6.15% in men older than 85 years [16]. Of note, only one of these studies required the confirmation of myocardial uptake by SPECT, which offers the advantage of better discriminating myocardial retention from blood pooling, which can lead to false-positive results on planar images [17]. Of note, the presence of myocardial uptake does not directly imply the presence of CA, which is characterized by abnormal findings on echocardiography and cardiac magnetic resonance. The careful evaluation of the clinical presentation of patients is essential for an operational interpretation of the scintigraphy image and to assess the need for further investigations such as CMR, tissue sampling for histological analysis or genetic investigation for TTR mutations. In case of complex clinical cases or unexpected results, multidisciplinary management and discussions are valuable resources for optimizing patient care [18,19].

In a series of 56 cases, Porcari et al. found diffuse amyloid deposits in 43% of hearts from unselected subjects aged >75 years who underwent autopsies, evenly distributed among ATTR-CA and AL-CA. CA patients more frequently had a history of HF, atrial fibrillation and LVH, and CA was the main cause of death in 14% of patients. Furthermore, no patient received a diagnosis of CA despite the presence of red flags in patients with histologically proven CA [20] (Table 1).

Moreover, the increasing number of incidental diagnoses posed a dilemma, especially in the era of tafamidis, as patients are often elderly (>80 years), with multiple comorbidities and limited life expectancy. The threshold for justifying treatment initiation with expensive medications in this population is a grey area, also considering that in the ATTR-ACT study, a survival benefit emerged after approximately 18 months of tafamidis [9].

## 3. Prevalence of CA in Different At-Risk Scenarios

- Carpal tunnel syndrome and CA

CTS is the most common compression syndrome of a peripheral nerve, mainly affecting patients older than 50 years, with a lifetime prevalence of CT release of about 3.1% in the general population [21]. The association between CTS and systemic amyloidosis has been widely investigated, with the main case series reporting a high prevalence in ATTR amyloidosis, ranging from 15% to 60% [22,23,24]. Moreover, CTS can be the first sign of ATTR amyloidosis and a definite red-flag during CA screening, given that patients with ATTR-CA may present with symptoms of bilateral CTS 5 to 15 years prior to their cardiac impairment [25]. Thus, CTS can be considered an early disease marker for cardiovascular (CV) outcomes, consistent with the future development of ATTR-CA, as proposed by Fosbøl et al. [25]. Similarly, observations by Porcari et al. supported the role of clinical phenotyping in patients with CTS, identifying those with unexplained LVH as a group at a higher risk of new-onset HF and CA during follow up [26]. However, a non-negligible percentage of patients present with CA already at the time of surgery. Zegri-Reiriz et al. reported a CA prevalence in 1.2% of a cohort of 233 patients aged >60 years with previous CTS surgery, which increased among those with LVH and bilateral CTS (5.5%) and in subjects without occupational risk factors for CTS (13.6%) [27]. In the ACTUAL study, in a cohort of 53 male patients (median age 73 years) with bilateral CTS screened for CA, the prevalence of disease was 4%, with a peak of 33% in the subgroup with LVH [28].

Furthermore, Milandri et al. compared the frequency of CTS between a cohort of patients with CA and subjects from the general population, finding the highest CTS prevalence in ATTR-CA subjects (20.3%) [29]. CTS standardized incidence rates were markedly elevated in men > 80 years with ATTR amyloidosis, with similarly distributed ATTRv and ATTRwt forms. Moreover, the risk of developing CA over time was greater in patients with CTS, with a time interval of 5–9 years [29] (Table 1).

Given the high prevalence of CTS in ATTR-CA patients and its role as a marker of early disease and increased risk of CV events, patients undergoing CT surgery should be carefully assessed in presence of the following characteristics: bilateral CTS, absence of occupational factors, need for multiple surgeries to relieve symptoms, concomitant lumbar spine stenosis or spontaneous rupture of the biceps tendon and the presence of unexplained LVH. The recognition of these phenotypes at a higher risk of underlying CA and the onset of CTS several years before the development of CA offer the possibility to achieve an early ATTR-CA diagnosis, which is a key element in the era of disease-modifying therapies (Table 1). It would be desirable in the future that patients with scheduled CTS (mostly if bilateral or relapsing) surgery undergo cardiological evaluation, including echocardiography, to assess the presence of red flags of CA. Future dedicated studies are required.

b. Heart Failure and CA

In developed countries, the prevalence of HF is estimated to be around 1–2% in adults, increasing with ageing from 1% (<55 years) to >10% (≥70 years) [30]. In recent years, HFpEF has become the leading form of HF, being found in up to 50% of patients with HF [31]. CA is an increasingly recognized cause of HF and mortality [32], but it is still underdiagnosed [30].

Most epidemiological data derive from historical post-mortem studies that reported a disarming prevalence of concealed cardiac amyloidosis in 25% of unselected adults over 80 years old and in about 32% of patients over 75 years old with HFpEF [33].

Other historical post-mortem investigations on heterogeneous cohorts reported CA in about 20–25% of patients older than 80 years of age [20]. In a recent autopsy study conducted in patients with an ante-mortem diagnosis of HFpEF without clinically apparent amyloid and control subjects, Mohammed et al. found CA in 17% of HFpEF patients (mostly ATTR-CA) and in 5% of the control. Adjusting for age at death and sex, ATTRwt was more common in patients with HFpEF than in the controls [34]. Current data suggest that ATTRwt amyloidosis may be responsible for as many as 30% of HFpEF cases in patients over 75 years [35]. Administrative data from Medicare Beneficiaries in the United States of America demonstrate that the prevalence of CA increased from 18 per 100,000 person years to 55.2 per 100,000 person years and the incidence of CA increased from 8 patients to 16.6 patients per 100,000 person years from 2000 to 2012, particularly among male patients over 65 years old presenting with new-onset HF [35].

A prospective study, using bone scintigraphy as screening tool for ATTR-CA in patients with HFpEF, showed that ATTRwt amyloidosis accounts for 13% of patients over 60 years with LVH hospitalized for HFpEF (Departments of Cardiology and Internal Medicine), with a mean age of 86 years [36]. A similar prevalence was found in the study by Lindmark et al., which reported a prevalence of 15% ATTRwt-CA in 86 patients with HFpEF [37].

In another study, AbouEzzedine et al. reported a prevalence of ATTR-CA of about 6% in a population of 286 patients with HFpEF, using bone scintigraphy, monoclonal protein and biopsy when needed [38]. In a study using only myocardial histology for diagnosis, CA was diagnosed in 14% of the 108 HFpEF patients (7 ATTRwt, 4 ATTRv, 3 AL and 1 AA) [39]. Another study using scintigraphy demonstrated that among Afro-Caribbean patients admitted with HF, ATTRv-CA was identified in 10% of cases to be attributable to the Val122Ile mutation [40].

A large multinational clinical trial is ongoing (NCT 04424914) aiming at enrolling 2000 participants with HFpEF in order to determine the global prevalence of ATTR-CA. HFpEF patients older than 60 years and with LVH will be screened for ATTR-CA by means of bone scintigraphy. This study will offer an important and more reliable update of the prevalence of ATTR-CA in this high-risk condition.

Prevalence of ATTR-CA was also evaluated in patients with HFrEF/HFmrEF; Lopez-Sainz et al. reported a prevalence of approximately 11% in HFrEF/HFmrEF patients [41], while Goland et al. found ATTR-CA in 9.3% of 75 HF patients with reduced LVEF [42] (Table 1).

In conclusion, HFpEF is a heterogeneous syndrome, with several underlying etiologic and pathophysiologic factors, among which CA is emerging. An epidemiological approach to patients with HFpEF must lead us to evaluate the several following parameters in addition to the presence of signs and symptoms of HF and the LVEF, in order to formulate the diagnosis of CA: the presence of unexplained LVH, the discrepancy between QRS voltage and LVH, echocardiographic red flags of infiltrative cardiomyopathy, troponin release, the increase in out-of-proportion natriuretic peptides compared to the degree of HF severity, hypotension or poor tolerability of antihypertensive therapy and the extra-cardiac context (e.g., CTS). Historically, HFpEF represents a syndrome without effective medical therapy on hard endpoints such as survival. The value of an etiological characterization of the disease lies in the ability, evident in the case of ATTR amyloidosis, to develop for the first time drugs that improve the prognosis of patients with HFpEF.

c. Aortic stenosis and CA

CA shares several common features with AS, such as LV concentric hypertrophy, impairment of LV diastolic function and, ultimately, HF. ATTR is the most prevalent type of CA associated with AS. The prevalence of both conditions increases with age, so their coexistence is not uncommon in the elderly [43] and can complicate the diagnosis and therapeutic management of the patients. Cardiac deposition of amyloid can infiltrate any cardiovascular structure, as well as the valves, whose thickness often becomes the “red flag” that raises the suspicion of CA. Moreover, aortic valve infiltration by amyloid substances may contribute to the initiation and progression of AS [44,45]. In the last few years, many studies investigated the combination of these two diseases. In 2016, Treibel et al. reported an ATTR-CA prevalence of 6% among patients with AS aged > 65 years undergoing surgical aortic valve replacement (SAVR) [46]. Similarly, Singal et al. performed PYP scintigraphy in 32 patients older than 65 years who were symptomatic with severe AS and underwent SAVR, revealing a significant radiotracer uptake (Perugini grade II or III) in three (9.4%) of them [47]. Interestingly, the EMB did not show amyloid deposition in the interventricular septum (IVS) but only in the aortic valves, suggesting isolated valvular amyloid deposition [47].

Some other studies investigated the prevalence of CA in patient with severe AS undergoing transcatheter aortic valve replacement (TAVR). Castaño et al. described a prevalence of 16%, mostly associated with a phenotype of low-flow low-gradient severe AS concomitant with a mildly reduced ejection fraction [48]. More recently, Scully [49] and Nitsche [50] reported CA in, respectively, 13.9% and 11.8% of two cohorts of patients using TAVR, which was studied by DPD bone scintigraphy, with only one of these affected by AL-CA [50]. Only one study also enrolled patients with moderate AS, reporting a concomitant presence of CA in 8% of these, with an average age for patients with CA higher than that of those with isolated AS [51]. Among those over the median age of 74 years, the prevalence of CA was 16%; in addition, after excluding women, the prevalence was 32% [51].

Importantly, AS and CA frequently harbor a low-flow, low-gradient pattern and thus require calcium scoring by computed tomography (CT) to confirm AS severity [43].

Interestingly, all the studies reported that patients with AS associated with CA had a poorer outcome than those with AS alone, with an increased risk of HF, mortality, and treatment futility with aortic valve replacement. These findings suggest the importance of non-invasive systematic CA screening in male patients >75 years presenting with severe AS, especially if presenting with a low-flow low-gradient AS pattern, also because, given the high surgical risk of patients with AS and concomitant CA, TAVR may be preferred to SAVR in this subgroup. In their paper, Nitsche et al. reported that the independent predictors of the presence of CA in AS patients are a longer QRS duration and a lower voltage/mass ratio and history of CTS; moreover, patients with AS-amyloidosis had a lower prevalence of coronary and peripheral artery disease, a significantly decreased functional capacity as measured by a 6 min walking test (6-MWT) and significantly elevated cardiac biomarkers (NT-proBNP and hsTnT) [50]. Following echocardiographic assessment, patients with AS-CA had slightly lower gradients, although no significant difference was found in absolute or indexed aortic valve area, and exhibited worse cardiac remodeling with greater LVH and worse diastolic dysfunction; the global longitudinal strain (GLS) was not different, but, as expected, relative apical sparing was more pronounced in AS-CA [50] (Table 1). The best therapeutic approach in AS-CA patients has yet to be established, particularly now that new drugs, such as tafamidis, are entering clinical practice. The decision making for the type (SAVR, TAVR, tafamidis, chemotherapy) and timing of treatment in symptomatic AS-CA patients is challenging and they should be carefully evaluated by the Heart Team. TAVR is often preferred to SAVR given that these patients are generally at high surgical risk and show a higher risk for structural valve deterioration following biological AVR [43]. In patients with moderate AS and CA, it may be worthwhile to consider the impact of agents targeting TTR on the rate of progression of AS severity. Additionally, the early removal of the hemodynamic stress imposed by the stenosed valve (early AVR) may delay the progression of TTR amyloid deposition in severe AS patients with isolated valvular amyloidosis. However, further studies are needed in order to investigate the best therapeutic approach in AS-CA patients

d. Hypertrophic Cardiomyopathy misdiagnosis

Patients diagnosed late in life with hypertrophic cardiomyopathy (HCM) represent a group at higher risk of CA, as this is when the incidence of CA is significantly increased and the misdiagnosis of these two conditions can occur [52].

HCM has an estimated prevalence of 1 in 500 to 1 in 5000 of the general population [30]. Once considered a disease of the young, HCM is now increasingly diagnosed in older middle-aged adults with near-normal life expectancy. Recent data from the International SHaRe registry (Sarcomeric Human Cardiomyopathy Registry), including 7286 HCM patients diagnosed between 1961 and 2019, found that the mean age at HCM diagnosis has been steadily increasing in the years from an average of 40 years old before 2000 to 51 years old after 2010. The rate of diagnoses for patients > 60 years increased from 9.2% before 2000 to 31.8% after 2010, and the prevalence of patients diagnosed at over 70 years reached 10.7% after 2010. Older patients were most frequently affected by sporadic forms of the disease and a more frequent genotype-negative status [53].

In a study enrolling 343 consecutive patients referred with an initial diagnosis of HCM at ≥40 years of age, CA was the most common HCM mimic, affecting 9% of the study population. Prevalence of CA linearly increased with the age of HCM diagnosis, ranging from 1% at ages 40–49 years to 26% above 80 years, with the large majority of cases being ATTR-CA [54].

A study using both cardiac magnetic resonance and bone scintigraphy, found a prevalence of 27% of ATTR-CA in a population of 114 patients with LVH at a mean age of 72 years [55].

In a prospective study, among 298 patients with unexplained LVH initially diagnosed as HCM, Damy et al. found a 5% prevalence of ATTRv-CA, using genetic testing to search for a TTR mutation and bone scintigraphy and CMR to provide clinical evidence of the mutation; all participants were older than 62 years. This study did not assess the presence of ATTRwt-CA [56].

Moreover, cases of ATTR-CA patients with outflow tract obstruction at rest or during stress have been reported [57,58] and ATTR-CA was diagnosed histologically in about 1% of patients referred for surgical myectomy [59] (Table 1).

Based on these findings, the potential for an HCM misdiagnosis and missed CA diagnosis in these patients should be carefully considered and they highlight the need for a systematic assessment of CA red flags in patients referred to HCM centers at ages > 50–60 years [54].

## 4. Challenges in Diagnosis and Treatment in CA

- Do not forget al. Cardiac Amyloidosis

The importance of early diagnosis of AL-CA cannot be stressed strongly enough, given that, if untreated, the median survival from the onset of HF is approximately 6 months [60], but modern therapies can put the disease into a prolonged remission and extend life by many years [61]. Thanks to the availability of proteosome-inhibiting agents, specifically bortezomib, which is usually combined with dexamethasone and low-dose cyclophosphamide, the prognosis of patients with AL-CA has considerably improved [60]. For these reasons, ruling out the disease can be considered an absolute clinical need. This can be achieved by measuring the proportion of kappa:lambda light chains with the serum free light chain assay, and testing for the immunofixation electrophoresis of serum and urine, which has a pivotal role in the Gillmore algorithm [5,62]. The active screening for AL-CA with this simple test has led to many early diagnoses of the disease in the last few years.

Unlike ATTR-CA, it seems that the incidence of AL-CA has not changed significantly in the past years. Kyle et al. reported an incidence rate of AL amyloidosis from 1990 through 2015 of 1.2 per 100,000 person years in the Olmsted County, with rates similar across the decades of 1990–1999, 2000–2009, and 2010–2015 at 1.1, 0.9, and 1.6 per 100,000 person years, respectively, with no suggestion of an increase [63]. Similar findings were reported in two Italian studies, enrolling about 600 patients each, both showing a rapid and marked increase in the number of new diagnoses of ATTR-CA over the last decade, with only 20–30% of new diagnoses being AL-CA, and stable during the years [64,65]. All the studies show that there is a slight male predominance of AL-CA, and the disease generally presents from the fifth to seventh decade, although it may occur at all ages from the fourth decade onward [60]. Given the poor prognosis of AL-CA, it is important that, once CA is suspected, a clonal dyscrasia is excluded using all the following tests: serum free light chain (FLC) assay, serum (SPIE), and urine (UPIE) protein electrophoresis with immunofixation, whose combination has a sensitivity of 99% for identifying abnormal pro-amyloidotic precursor in AL amyloidosis [66,67]. Remarkably, a monoclonal gammopathy of undetermined significance (MGUS) might be found in up to 40% of ATTR-CA [68] and, on the other hand, about 30% of patients with EMB-confirmed AL-CA show a grade 2 or 3 cardiac uptake on planar images of bone tracers scintigraphy [5,66]. Diagnosis of CA, in this case, requires histology with amyloid typing, usually via EMB [66]. Nevertheless, an extended hematological workup must always be required.

b. When chronic inflammatory or infectious diseases causes Amyloidosis: Amyloid A (AA) Amyloidosis

Previously known as secondary or reactive amyloidosis, systemic AA amyloidosis is a long-recognized serious complication of some chronic inflammatory and infectious diseases (such as tuberculosis, osteomyelitis, rheumatoid arthritis, Familial Mediterranean Fever, vasculitis) caused by the extracellular deposition of the soluble acute-phase reactant serum amyloid A (SAA) protein, produced by the liver [69]. Available knowledge indicates that the frequency of heart involvement in AA amyloidosis is about 5%; therefore, significant clinical cardiac manifestations are rare. However, it has been shown that SAA may be deposited in cardiac tissue [70], leading to severe ventricular wall thickening and subsequent stiffness resulting in a restrictive pattern as well as ventricular arrhythmias as major cardiac manifestations [71].

In developed countries, the incidence of AA amyloidosis is low since this disorder only occurs as a long-term complication of rather severe chronic inflammatory disorders that in turn are mostly well-managed but it can be suspected in the presence of renal impairment with proteinuria, hepatomegaly and gastrointestinal problems [66]. However, no convincing non-invasive imaging findings have been described so far regarding the detection of cardiac involvement in cases of AA amyloidosis and EMB remains the gold-standard for the final diagnosis.

c. What is the role of endomyocardial biopsy?

Endomyocardial biopsy (EMB) has been progressively limited as a diagnostic tool, but it still represents an important technique to reach a definite as well as an etiological diagnosis in controversial cases [72]. The analysis of the data from the ‘Cardiac Amyloidosis Registry’ of Trieste from January 1990 to December 2020 clearly shows that patients in the contemporary cohort (67% ATTR-CA) are diagnosed more frequently using the non-invasive approach compared to those in the historical cohort [73]. In some cases, scintigraphy shows cardiac uptake and at least one of the monoclonal protein tests is abnormal; ATTR-CA with concomitant MGUS or the coexistence of both AL and ATTR-CA is possible in this scenario. EMB, performed in specialized centres, can identify amyloid deposits after Congo red staining, define the amyloid fibril protein using mass spectrometry, immunohistochemistry, or immunoelectron microscopy and show the possible presence of inflammatory components and/or fibrosis. Therefore, EMB may be relevant in selected cases for assessing the correct diagnosis and providing significant clues for both the diagnosis and the understanding of this rather complex and heterogenous disease [66].

d. Impact of the advances in epidemiology on the recognition and diagnostic work-up of patients at suspicion of CA

The latest advances in the knowledge of the epidemiology of CA have contributed to increasing the awareness in the scientific community, changing the approach to the disease. The abovementioned studies clearly demonstrated that there are a series of clinical scenarios and clues that should raise suspicions of CA, which include the following: HFpEF, whose CA represents a specific subtype and is potentially treatable; HCM, when diagnosed in patients ≥ 40 years of age; AS, above all with an LF-LG pattern; and CTS, when bilateral and without occupational risk factors. When facing these particular “high-risk” phenotypes, CA has to be systematically searched and excluded, thus facilitating an early and correct diagnosis and indicating the choice of the most appropriate therapeutic management. A pivotal role is played by echocardiographic laboratories, given that CA typically appears within a constellation of echocardiographic findings, the so-called “red-flags”, that, when associated with suggestive signs and symptoms, can properly direct the subsequent diagnostic work-flow. Moreover, there are areas of uncertainty that still have to be addressed, such as how to manage the unclear scenarios of cardiac involvement in asymptomatic patients or positive bone scintigraphy without a clear echocardiogram or CMR findings.

Once the correct diagnosis is achieved, the cardiologist has to face many grey areas in CA management, where there are still significant knowledge gaps that none of the recent national or international scientific societies consensus documents have addressed [74]. Some of the most interesting questions concern how to monitor disease progression, how to stratify patient risk, which patients should receive a pacemaker or undergo cardiac resynchronization, which patients need an ICD in primary prevention, if there are patients with a sinus rhythm that should receive anticoagulants and how to choose between disease-modifying therapies. Tafamidis is currently the only approved treatment for patients with ATTRwt-CA or ATTRv-CA without polyneuropathy but the prescription of this expensive medication has to be carefully evaluated and justified because real-world ATTR-CA populations do not exactly match those enrolled in clinical trials in which treatment with tafamidis has proven to be effective. Gene-silencing agents, such as patisiran and inotersen, have entered clinical practice. They are agents capable of silencing the TTR gene by degrading TTR mRNA and reducing the concentration of circulating TTR. Patisiran is approved for the treatment of patients with ATTRv polyneuropathy with or without ATTRv-CA, while inotersen is recommended in patients with ATTRv polyneuropathy without cardiac involvement. Therefore, tafamidis should be generally considered the agent of choice in ATTR-CA patients with a reasonable expected survival while patisiran could be considered in patients with ATTRv polyneuropathy and cardiac involvement [66,75]. Another issue that needs to be solved is when to search for a gene mutation in family members and how mutation carriers should be managed. Indeed, if a consensus is reached on the fact that, once ATTR-CA has been recognized, genetic counseling and testing should be recommended, how best to manage first degree relatives is not clear. In order to address all these issues and better manage CA patients, further investigations are needed, thus making the disease a fascinating and rapidly evolving field of study.

**Table 1:** Screening studies on CA in different settings.

Authors	Year	Indication to Screening	Setting	Population (n)	Mean/ Median Age	CA Prevalence (%)	Diagnostic Algorithm
Longhi S. et al. [13]	2014	Bone scan for non-cardiac reasons	Scintigraphy with bone tracers	12,400	74	0,4	Scintigraphy with bone tracers (EKG, echocardiography, EMB in selected patients with scintigraphy+)
Bianco M. et al. [14]	2021	Bone scan for any reasons	Scintigraphy with bone tracers	4228	N/A	0,5	Scintigraphy with bone tracers
Mohamed-Salem L. et al. [15]	2018	Bone scan for non-cardiac reasons, ≥75 years	Scintigraphy with bone tracers	1114	81	2,8	Scintigraphy with bone tracers
Cuscaden C. et al. [16]	2021	Bone scan for non-cardiac reasons	Scintigraphy with bone tracers	6918	N/A	0,2	Scintigraphy with bone tracers
Zegri-Reiriz I. et al. [27]	2019	CTS surgery, ≥60 years, LV wall thickness ≥ 12 mm	CTS	101	69	3	Scintigraphy with bone tracers, monoclonal protein, biopsy when needed
Vianello PF. et al. [28]	2021	Bilateral CTS surgery in male patients	CTS	53	73	4	Scintigraphy with bone tracers, monoclonal protein
Tanskanen M. et al. [33]	2008	Autopsy > 85 years	Autopsy	256	N/A	25	Histology
Mohammed S. et al. [34]	2014	Autopsy in HFpEF patients/control subjects	Autopsy/HFpEF	109 (HFpEF)/131 (Control)	76 (HFpEF)/69 (Control)	17 (HFpEF)/5 (Control)	Histology
Porcari A. et al. [20]	2021	Autopsy ≥ 75 years	Autopsy	56	86	43 (diffuse Amyloidosis in the LV: 29%)	Histology
Gonzalez-Lopez E. et al. [36]	2015	HF hospitalization, ≥60 years, LV wall thickness ≥ 12 mm	HFpEF	120	86	13	Scintigraphy, monoclonal protein, biopsy when needed
Lindmark K. et al. [37]	2021	HF clinic, LV wall thickness ≥14 mm	HFpEF	86	77	15	Scintigraphy, monoclonal protein, biopsy when needed
AbouEzze ddine OF. et al. [38]	2021	HF, LVEF ≥ 40%, LV wall thickness ≥ 12 mm, ≥60 years	HFpEF	286	78	6	Scintigraphy, monoclonal protein, biopsy when needed
Hahn VS. et al. [39]	2020	HFpEF	HFpEF	108	66	14	Histology (EMB)
Merlo M. et al. [8]	2022	LV wall thickness ≥ 12 mm, LVEDVi ≤ 85 mL/m^2^, LVEF ≥ 50%, ≥55 years, at least 1 echocardiographic red flag of CA	HFpEF/LVH	217	75	29 (Apical sparing or a combination of ≥2 other echocardiographic red flags, excluding interatrial septum thickness, provided a diagnostic accuracy > 70%)	Scintigraphy, monoclonal protein, extra-cardiac histology, genetic test. CMR and cardiac histology (EMB) in selected patients, if needed.
Dungu JN [40]	2016	Afro-Caribbeans patients admitted with HF	HF	211	71	11 (8,5% with Val122Ile mutation)	CMR, scintigraphy, monoclonal protein, biopsy when needed, genetic test
Lopez-Sainz A. et al. [41]	2019	HF hospitalization, LVEF < 50%, >60 years, LV wall thickness ≥ 12 mm	HFrEF/HF mrEF	28	78	11	Scintigraphy, monoclonal protein, biopsy when needed
Goland S. et al. [42]	2021	Unexplained LV systolic dysfunction	HFrEF/HF mrEF	75	65	9	Scintigraphy, monoclonal protein, biopsy when needed
Treibel TA. et al. [46]	2016	AS referred to SAVR, >65 years	AS	146	71	4	Histology (intraoperative biopsy)
Singal AK. et al. [47]	2021	AS referred to SAVR, >65 years	AS	32	70	9 (no Amyloid in IVS biopsy, 72% in the aortic valve)	Scintigraphy, histology
Castano A. et al. [48]	2017	AS referred to TAVR	AS	151	84	16	Scintigraphy, monoclonal protein
Scully P. et al. [49]	2018	AS referred to TAVR, >75 years	AS	101	86	14	Scintigraphy, monoclonal protein
Nitsche C. et al. [50]	2021	AS referred to TAVR	AS	407	83	12	Scintigraphy, monoclonal protein
Cavalcante JL. et al. [51]	2017	Moderate/severe AS referred to CMR	AS	113	70	8	CMR (suspected CA)
Maurizi N. et al. [54]	2019	Initial diagnosis of HCM	HCM	343	60	9	Genetic test, if no TTR mutations but ≥1 CA red flag, monoclonal protein, abdominal fatbiopsy and/or scintigraphy and ApoAI sequencing
Cariou E. et al. [55]	2017	LV wall thickness ≥12 mm	HCM	114	72	27	Scintigraphy, monoclonal protein, CMR
Damy T. et al. [56]	2016	Initial diagnosis of HCM	HCM	298	62	5	*TTR* gene testing, then scintigraphy, CMR, biopsy
Helder MRK. et al. [59]	2014	HCM underwent septal myectomy	HCM	1714	N/A	1	Histology

Legend: ApoAI, apolipoprotein AI; AS, aortic stenosis; CA, cardiac amyloidosis; CMR, cardiovascular magnetic resonance; CTS, carpal tunnel syndrome; LVEDVi, indexed left ventricular end-diastolic volume; EMB, endomyocardial biopsy; EKG, electrocardiogram; HCM, hypertrophic cardiomyopathy; HF, heart failure; HFmrEF, heart failure with mildly reduced ejection fraction; HFpEF, heart failure with preserved ejection fraction; HFrEF, heart failure with reduced ejection fraction; IVS, interventricular septum; LV, left ventricle; LVEF, left ventricle ejection fraction; LVH, left ventricular hypertrophy; N/A, not available; SAVR, surgical aortic valve replacement; TAVR, transcatheter aortic valve replacement; TTR, transthyretin.

The figure reveals the prevalence of CA in different screening studies, grouped together according to the different scenarios. Information from the original studies is reported in Table 1.

Legend: CA, cardiac amyloidosis; HCM, hypertrophic cardiomyopathy; HFmrEF, heart failure with mildly reduced ejection fraction; HFpEF, heart failure with preserved ejection fraction; HFrEF, heart failure with reduced ejection fraction.

## 5. Conclusions

CA is increasingly diagnosed, with an epidemiology that is rapidly evolving but remains largely unknown. Specific clinical settings have been recognized as strongly associated with CA but further studies are needed to better characterize these populations, mainly to stratify patient risk and evaluate which patients are ideal candidates for the newly available disease-modifying therapies.

## Data Availability

Not applicable.
